# Trivalent Combination Vaccine Induces Broad Heterologous Immune Responses to Norovirus and Rotavirus in Mice

**DOI:** 10.1371/journal.pone.0070409

**Published:** 2013-07-26

**Authors:** Kirsi Tamminen, Suvi Lappalainen, Leena Huhti, Timo Vesikari, Vesna Blazevic

**Affiliations:** Vaccine Research Center, University of Tampere School of Medicine, Tampere, Finland; Pasteur Institute of Shanghai, Chinese Academy of Science, China

## Abstract

Rotavirus (RV) and norovirus (NoV) are the two major causes of viral gastroenteritis (GE) in children worldwide. We have developed an injectable vaccine design to prevent infection or GE induced with these enteric viruses. The trivalent combination vaccine consists of NoV capsid (VP1) derived virus-like particles (VLPs) of GI-3 and GII-4 representing the two major NoV genogroups and tubular RV recombinant VP6 (rVP6), the most conserved and abundant RV protein. Each component was produced in insect cells by a recombinant baculovirus expression system and combined *in vitro*. The vaccine components were administered intramuscularly to BALB/c mice either separately or in the trivalent combination. High levels of NoV and RV type specific serum IgGs with high avidity (>50%) as well as intestinal IgGs were detected in the immunized mice. Cross-reactive IgG antibodies were also elicited against heterologous NoV VLPs not used for immunization (GII-4 NO, GII-12 and GI-1 VLPs) and to different RVs from cell cultures. NoV-specific serum antibodies blocked binding of homologous and heterologous VLPs to the putative receptors, histo-blood group antigens, suggesting broad NoV neutralizing activity of the sera. Mucosal antibodies of mice immunized with the trivalent combination vaccine inhibited RV infection *in vitro*. In addition, cross-reactive T cell immune responses to NoV and RV-specific antigens were detected. All the responses were sustained for up to six months. No mutual inhibition of the components in the trivalent vaccine combination was observed. In conclusion, the NoV GI and GII VLPs combination induced broader cross-reactive and potentially neutralizing immune responses than either of the VLPs alone. Therefore, trivalent vaccine might induce protective immune responses to the vast majority of circulating NoV and RV genotypes.

## Introduction

Acute gastroenteritis (AGE) is a leading cause of morbidity and mortality in children all over the world [1]. Viruses are responsible for a significant number of AGE cases and two leading agents for viral gastroenteritis are rotavirus (RV) and norovirus (NoV) [1]. Following the introduction of live RV vaccines Rotarix® (GlaxoSmithKline plc, UK) and RotaTeq® (Merck & Co., Inc., USA) into national immunization programs, NoV’s epidemiological importance is rising and in some countries NoV has already overtaken RV as the most important cause of pediatric AGE [2–5].

Development of a NoV vaccine is underway [6–8]. Since the cultivation of NoVs has not been successful [9], the main direction in vaccine development has been the use of non-live NoV virus-like particles (VLPs), which mimic the structure and the antigenic properties of the native NoVs [10]. These VLPs are constructed of the core protein VP1, which self-assembles into VLPs when produced *in vitro* [10]. An additional challenge in the NoV vaccine development is the high genetic variation of NoVs [11]. The major NoV genogroups infecting human beings are genogroup I (GI) and genogroup II (GII) with at least 25 different genotypes belonging in these genogroups [11]. There is a great molecular variation inside the genotypes themselves and the driving force in the evolution seems to be herd immunity [12]. For over two decades the most prevalent NoV genotype has been GII-4, currently accounting for over 80% of all NoV cases [13,14]. There is some immunological cross-reactivity between GI and GII genogroups [15] but no protective immune responses across genogroups in humans have been observed [16]. It has been suggested that a broadly effective NoV vaccine should be a combination of at least two genotypes; one from each of the major genogroups [17–19].

RV annually accounts for ~450 000 deaths in children under 5 years of age, the majority of the deaths taking place in developing countries [20]. Since the introduction of the two live-attenuated RV vaccines, the cases of RV-caused AGE have decreased dramatically [5,21,22]. Despite the efficacy of RV vaccines, there are still certain limitations associated with both of these vaccines. The introduction of the vaccines into developing countries has been challenging [23] and safety issues like increased risk of intussusception [24,25] and the reassortment of vaccine viruses in higher virulence [26,27] are concerns involved in the currently available live-attenuated RV vaccines.

RV has a double stranded RNA genome enclosed in the triple layered capsid [28]. VP7 forms a virion surface from which spike-like structures (VP4) extend outwards and are responsible for cell attachment [28]. The inner capsid consists of VP6, which is highly antigenic and the most conserved RV protein [28]. Although neutralizing antibodies targeted against VP4 and VP7 are most strongly associated with RV immunity [29], anti-VP6 antibodies and CD4+ T cells have also been suggested to play a role in the protection [30–33]. RV recombinant VP6 (rVP6) has the ability to form various assemblies *in vitro* [34] and these structures are considered the second-generation vaccine candidates for non-live RV vaccine development [35].

We have previously shown that a dual combination of NoV GII-4 VLPs and RV rVP6 tubules induced strong humoral immune responses without mutual inhibition when delivered parenterally into BALB/c mice [7]. In the present study we have included GI-3 VLPs as a representative of GI NoVs into the above combination in an attempt to broaden NoV-specific immune responses. Induction and long-term duration of NoV and RV-specific cell mediated immunity in addition to humoral immune responses was investigated. Our data indicates that the trivalent combination vaccine containing GII-4 VLPs, GI-3 VLPs, and rVP6 induces robust, long-lasting and broadly cross-reactive NoV and RV-specific cellular immune responses and antibodies with neutralizing abilities against both viruses.

## Materials and Methods

### Ethics Statement

The protocol for the study was approved by the Finnish National Animal Experiment Board (permission number ESLH-2009-06698/Ym-23). All the procedures performed on the animals were conducted according to the guidelines of the Finnish National Animal Experiment Board and all efforts were made to minimize animal suffering.

### Production and purification of NoV VLPs and rVP6

NoV GII-4 VLPs, GI-3 VLPs, GII-4 New Orleans (NO) VLPs, GII-12 VLPs, GI-1 VLPs, and RV rVP6 used for immunizations and/or as antigens in immunological assays were produced by a baculovirus-insect cell expression system and purified by sucrose gradients as previously described [7,36]. The reference strains for each genotype were determined according to the EMBL/Genbank classification and FBVE as the following: AF080551 (GII-4-1999), AF414403 (GI-3-2001), GU445325 (GII-4 New Orleans, GII-4 NO-2010), AJ277618 (GII-12-1998), AY502016.1 (GI-1-2001) and GQ477131 (RV G1P1A [8]-2007 derived VP6). The morphology, integrity, purity, *in vitro* antigenicity and protein concentration were determined for each protein as described previously [7,36].

### Cultivation of RVs in cell culture

The RV cultures used as antigens in the enzyme-linked immunosorbent assay (ELISA) and enzyme-linked immunosorbent spot (ELISPOT)-interferon-γ (IFN-γ) assays were propagated in an MA104 cell line (ATCC CRL-2378, LGC Standards, UK) as described by others [37]. In short, MA104 cells were infected with the human RV strains Wa (G1P1A [8]), SC2 (G2P2 [6]), BrB (G4P2 [6]), 69M (G8P4 [10]), L26 (G12P1B [4]), bovine WC3 (G6P7 [5]), and rhesus rotavirus (RRV, G3P5B [3]) and after observing the maximum cytopathic effect (3-4 days respectively), the viruses were collected and the VP6 protein amount in each culture was determined by capture ELISA using insect cell-derived rVP6 as an internal standard. The RV cell culture antigens were diluted in phosphate-buffered saline (PBS) to contain equal quantities of VP6 protein per each culture.

### Mice immunizations and sample collections

To determine the optimal amount of each antigen, three doses (3, 10 or 30 µg) of NoV GII-4 VLPs, GI-3 VLPs or RV rVP6 were administered intramuscularly (IM) to 7-week-old female BALB/c mice (Harlan laboratories, Horst, the Netherlands). The mice were immunized (5 mice/group) at study weeks 0 and 3 and euthanized at study week 5. After the optimal dose selection, naïve BALB/c mice (5 mice/group) were immunized in another set of experiments according to the above schedule with a single NoV GII-4 VLPs, GI-3 VLPs or RV rVP6 antigen (each at a 10 µg dose) or the trivalent combination (10 µg GII-4 VLPs + 10 µg GI-3 VLPs + 10 µg rVP6) and euthanized at study week 5. A group of mice receiving the trivalent combination vaccine (7 mice/group) were euthanized at study week 27 for the long-term follow-up of the immune responses. Negative control groups of mice (5-7 mice/group) received carrier only (PBS) and were terminated at week 5 or week 27. Blood samples were collected at study weeks 0 (pre-immune serum), 3, 4, 7, 12, 16, and 20 as previously described [38]. Whole blood, feces, lymphoid tissues and vaginal washes (VW) were collected at the time of euthanization. Serum was separated from the blood of each mouse and 10% (w/v) stool suspensions were prepared from group-wise pooled stools according to the published procedures [7]. Preparation of the cell suspensions and freezing of the splenocytes were conducted as described earlier [38]. VWs were collected by pipetting 2 × 125 µl of cold PBS into the vagina 4-5 times up-and-down, after which the VW were centrifuged at 12,000 × g for 10 minutes at +4^°^C and the supernatant stored at -20^°^C.

### NoV and RV-specific immunoglobulin (Ig) detection from serum and IgG avidity assay

ELISA used to measure antigen-specific IgG, IgG1, and IgG2a from serum is described in details elsewhere [7,38]. Briefly, 96-well half-area polystyrene plates (Corning Inc., Corning, NY) were coated with GII-4, GI-3, GII-4 NO, GII-12 or GI-1 VLPs (0.4-1.5 µg/ml) or rVP6 (0.8 µg/ml). For the detection of antibodies against various RV culture antigens (described above) the plates were precoated with rabbit anti-rotavirus polyclonal antibody (DakoCytomation, Glostrup, Denmark) at 1 µg/ml in PBS followed by the addition of RV cell culture antigens at VP6 antigen concentration of 0.1 µg/ml. The serum samples (at 1:200 dilution or 2-fold dilution series) from immunized and control mice were added to the plates and the bound antibody was detected with HRP conjugated goat anti-mouse IgG (Sigma-Aldrich, Saint Louis, MO), IgG1 (Invitrogen, Carsbad, CA) or IgG2a (Invitrogen) followed by the reaction with the OPD substrate (Sigma-Aldrich). The optical density (OD) was measured at 490 nm (Victor2 1420; PerkinElmer, Waltham, MA). The background signal from the blank wells (wells without serum) was subtracted from all of the OD readings at a plate. The cutoff value was calculated from the wells of negative control mice serum as mean OD + 3 × SD. A sample was considered positive if the net OD value was above the set cut-off and at least 0.100 OD. End-point antibody titers were defined as the highest dilution of serum giving an OD above the set cut-off value. A Th2/Th1 response ratio was calculated by dividing the end-point titer of IgG1 response with the corresponding IgG2a titer.

Serum IgG avidity was measured by ELISA as described above with an extra urea incubation step to remove the low avidity antibodies [39,40]. The avidity index was calculated as (OD with urea/OD without urea) × 100% and avidity index ≥ 50% was considered high avidity.

### NoV and RV-specific immunoglobulin (Ig) detection from mucosal samples and RV-specific IgA detection from serum

NoV-specific IgG content was tested from stool suspensions (10% suspension) with the ELISA as described above. RV rVP6-specific IgG and IgA in the stool suspensions and VWs and rVP6-specific IgA in serum were detected by sandwich ELISA as follows. The 96-well plate was first coated with rabbit anti-rotavirus polyclonal antibody (DakoCytomation, Glostrup, Denmark) at 1 µg/ml in PBS followed by the addition of rVP6 (1 µg/ml in PBS). After washing the unbound rVP6, 10% fecal suspensions (serially diluted from 1:5), VW samples (diluted 1:5 for IgG detection and 1:2 for IgA detection) or serum (diluted 1:2) were added and the plate was developed with 1:4000 diluted HRP conjugated goat anti-mouse IgG or IgA (both from Sigma-Aldrich) and OPD substrate.

### NoV VLP blocking assays

Saliva-based blocking assays were used as a surrogate neutralization assay for NoV [41] and the procedure is described in details elsewhere [38]. In brief, serum dilutions from immunized and control mice were pre-incubated with NoV VLPs (at concentrations 0.1-0.2 µg/ml) for 1 h at 37^°^C and added to secretor positive human saliva type A (for GII-4, GII-4 NO and GI-3 VLPs binding) or type O (for GI-1 VLP binding) coated 96-wells plates. VLPs lacking the serum were used for maximum binding of VLPs to the saliva. The VLPs bound to histo-blood group antigens (HBGAs) present in saliva were detected with NoV antibody positive human serum [40] and anti-human IgG-HRP (Invitrogen) following the OPD substrate development. The blocking index (%) was calculated as 100% – (OD wells with serum/OD wells without serum, maximum binding) × 100%.

### Inhibition of RV infectivity in vitro

The ability of mucosal and serum antibodies to abolish RV infectivity *in vitro* was determined by an ELISA-based RV antigen reduction neutralization assay (NELISA) as described by others [42,43] with slight modifications. Two-fold dilution series of group wise pooled and 1:10 diluted fecal samples, VWs and sera from immunized and control mice were mixed with Wa (G1P1A [8]) RV strain homologous to the immunizing rVP6 protein or RRV (G3P5B [3]) containing 125 focus-forming units (ffu). RV antibody positive human serum diluted from 1:10 was used as a positive assay control. After 1 hour incubation at +37^°^C the mixtures were overlaid to confluent MA104 cell monolayers in 96-well cell culture plates (Nunc, Roskilde, Denmark) following centrifugation for 60 min at 1000 × g. The virus inoculum was replaced with a culture medium containing trypsin (Sigma-Aldrich) at 4 µg/ml and the plates were incubated for 15 h at +37^°^C. After lysing the cells with a thaw freeze cycle the RV detection in duplicate samples was performed by a Ridascreen® kit (R-Biopharm AG, Darmstadt, Germany) according to the manufacturer’s instructions. A reduction in OD value greater than 60% compared with the positive control wells (trypsin activated RV without the test sample) was considered to indicate neutralization. Neutralizing titers were expressed as the highest sample dilution yielding neutralization.

### Detection of interferon-γ (IFN-γ) producing T cells

NoV and RV-specific T cell responses were analyzed by quantification of IFN-γ production from splenocytes by ELISPOT [38] with slight modifications. Ninety-six well MultiScreenHTS-IP filter plates (Millipore, Billerica, MA) were coated with monoclonal anti-mouse IFN-γ (Mabtech Ab, Nacka Strand, Sweden) at 5 µg/ml. After blocking the plates with 10% fetal bovine serum (FBS, Sigma-Aldrich) the antigens and the cells in the culture media (CM, RPMI-1640 supplemented with 100 U/ml penicillin, 100 µg/ml streptomycin, 50 µM 2-mercaptoethanol and 2 mM L-glutamine, all purchased from Sigma-Aldrich) and 5% FBS were added. NoV capsid-derived synthetic 15-mer peptides (Proimmune Ltd., Oxford, UK) identical to a published T-cell epitope of GII-4 (CLLPQEWVQHFYQEA, amino acids 461–475) [44] and corresponding peptides of GII-4 NO (CLLPQEWVQYFYQEA) and GII-12 (CLLPQEWIQHLYQES) were used at 5 µg/ml to stimulate individual mouse splenocytes (0.1x10^6^ cells/well) for NoV-specific INF-γ production. For detection of RV-specific IFN-γ producing cells, group-wise pooled splenocytes were stimulated with VP6 derived 18-mer peptide previously identified as a VP6-specific CD4+ T cell epitope (DGATTWYFNPVILRPNNV, amino acids 242-259) [45] at 5 µg/ml or RV cell culture antigens (Wa G1P1A [8], BrB G4P2 [6], WC3 G6P7 [5] and RRV G3P5B [3]) at a VP6 concentration of 0.5 µg/ml. Mock infected MA104 cell cultures were used as a negative control. Background control (cells with CM only) and cell viability control (cells stimulated with 10 µg/ml of Conacavalin A, Sigma-Aldrich) were added to each assay. The plates were incubated for 20 h at +37^°^C and 5% CO^2^ after which the cells were discarded and the plates were thoroughly washed with PBS. Biotinylated anti-mouse IFN-γ monoclonal antibody (Mabtech, 0.5 µg/ml in PBS / 0.5% FBS) was added and the plates incubated for 2 h at RT. After washing, 1:1000 diluted streptavidin-ALP (Mabtech) was added and the plates were incubated for 1h. The spots were developed with BCIP/NBT substrate (Mabtech) and the formation of color reaction stopped with tap water. The spots were counted by an ImmunoSpot® automatic CTL analyzer (CTL-Europe GmbH, Bonn, Germany) and the results are expressed as mean spot-forming cells (SFC) per 10^6^ splenocytes of duplicate wells.

### Statistical analyzes

A Mann–Whitney *U*-test was used to assess the statistical difference between non-parametric observations of two independent groups. Statistical analyses were done by IBM SPSS Statistics -software (SPSS Inc., Chicago, IL) version 19.0 and the statistical significant difference was defined as p ≤ 0.05.

## Results

### Morphology of NoV VLPs and RV rVP6 and formulation of the trivalent vaccine

**Figure 1:**
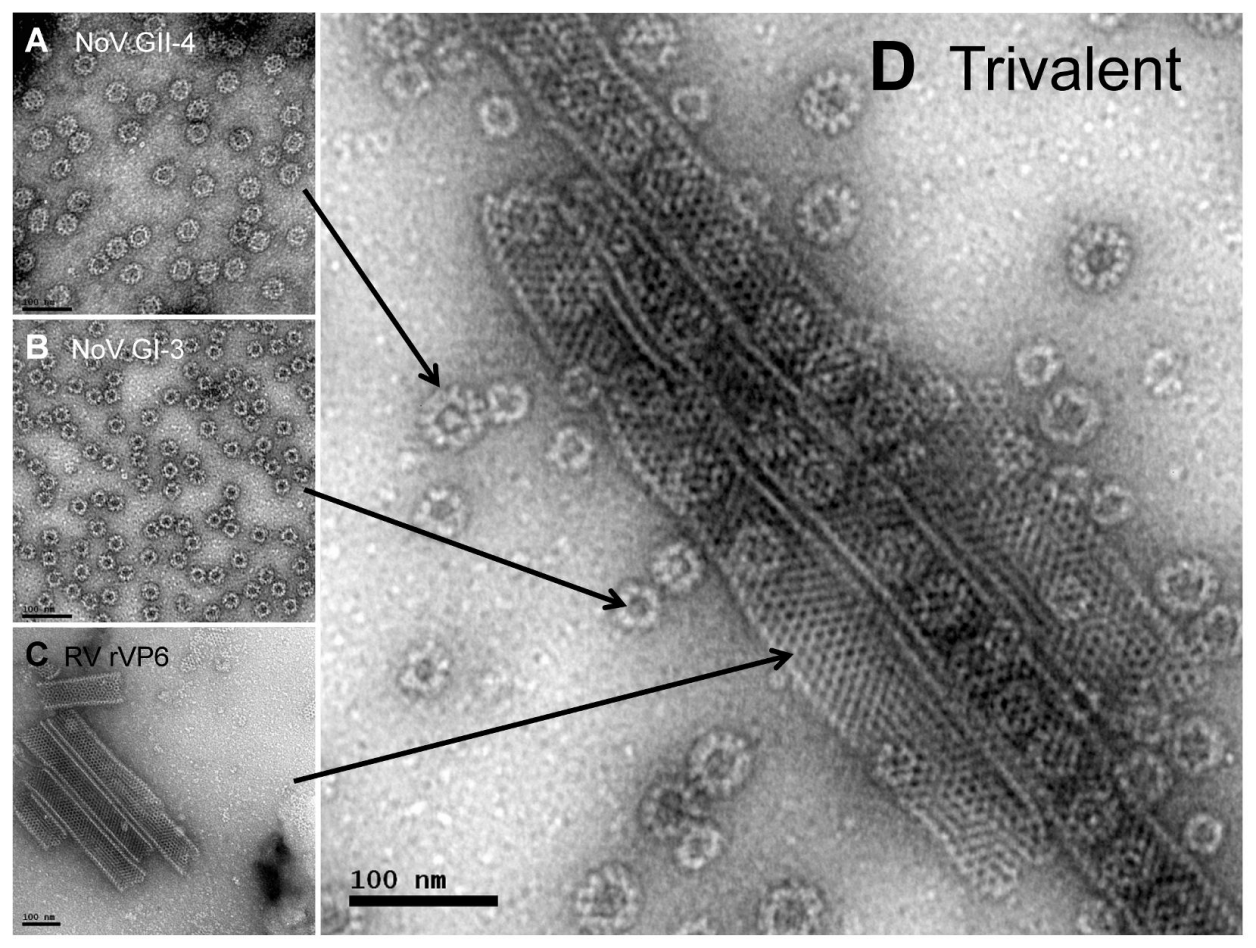
Electron microscopy images of the single antigens and the trivalent combination used to immunize BALB/c mice. Morphological assemblies of NoV GII-4 capsid (A), GI-3 capsid (B) and RV rVP6 (C) proteins, and the trivalent combination (1:1:1 of each antigen) of the structures depicted in panels A-C (D) were examined by transmission electron microscopy (TEM) using a FEI Tecnai F12 electron microscope (Philips Electron Optics, Holland) with 18,500 × magnification following negative staining with 3% uranyl acetate (UA), pH 4.6. The arrows represent each structure (A–C) in the trivalent assembly (D). Bar 100 nm.

The assembly conformations of NoV GII-4 VLPs, GI-3 VLPs and RV rVP6 were verified by transmission electron microscopy (TEM) as described previously [36]. As illustrated in Figure 1, recombinant BV-produced NoV VP1capsid proteins self-assembled into the GII-4 VLPs of ~38 nm (Figure 1A) and GI-3 VLPs (Figure 1B) of ~30 nm in diameter. RV rVP6 production resulted in conformation of VP6 trimers, which under neutral pH conditions (PBS, pH 7.4) assembled into tubular structures but also to a minor number of sheets (Figure 1C) [34]. The combination of both NoV VLPs and rVP6 in the ratio of 1:1:1 resulted in the trivalent formulation where the VP6 tubules were partly filled with the VLPs (Figure 1D).

### Dose response of single antigen immunizations

**Figure 2:**
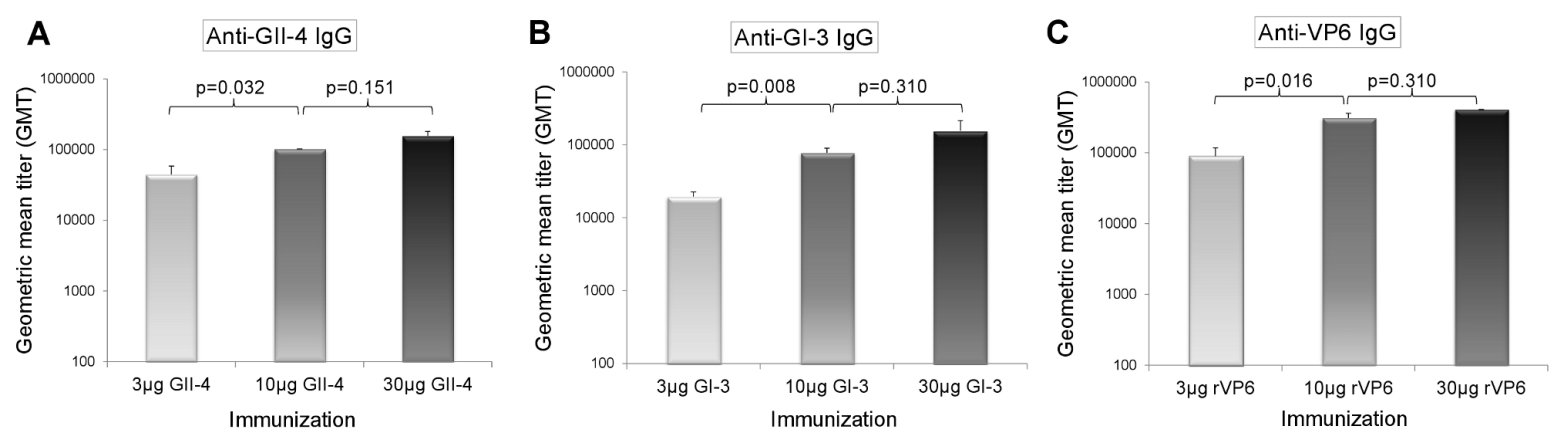
Antigen-specific serum IgG dose response. Mice were immunized twice at study weeks 0 and 3 with 3, 10 and 30 µg of single antigens and the geometric mean titers (GMTs) induced by GII-4 VLPs (A), GI-3 VLPs (B) and RV rVP6 (C) were measured in an ELISA. The error bars represent the standard error of the means. Statistical differences between any two experimental groups were determined by a Mann–Whitney *U*-test and the p-value ≤ 0.05 was considered a statistically significant difference.

The optimal amount of antigens to be used in the trivalent vaccine was pre-determined by a dose response study in BALB/c mice immunized with 3, 10 and 30 µg of NoV GII-4 VLPs, GI-3 VLPs or RV rVP6 as single antigens. The dose responses to each antigen were screened by measuring antigen-specific serum IgG antibody titers in ELISA. All three antigens induced robust systemic IgG responses in mice (Figure 2A–C). No significant difference (p>0.05) in the levels of IgG in the termination sera was detected between the groups immunized with 10 and 30 µg of the antigens, whereas a 3 µg dose raised the significantly lower IgG response to each of the antigens (p<0.05). Additional immunological assays including antigen-specific IgG avidity, IgG subtype ratio (IgG1/IgG2a), IgG cross reactivity, NoV VLPs blocking activity and intestinal antibody content confirmed the same result (data not shown). Therefore, the 10 µg dose for each antigen was used in the further immunogenicity studies described below.

### Magnitude and avidity of antigen-specific IgG responses and IgG subtype balance

**Figure 3:**
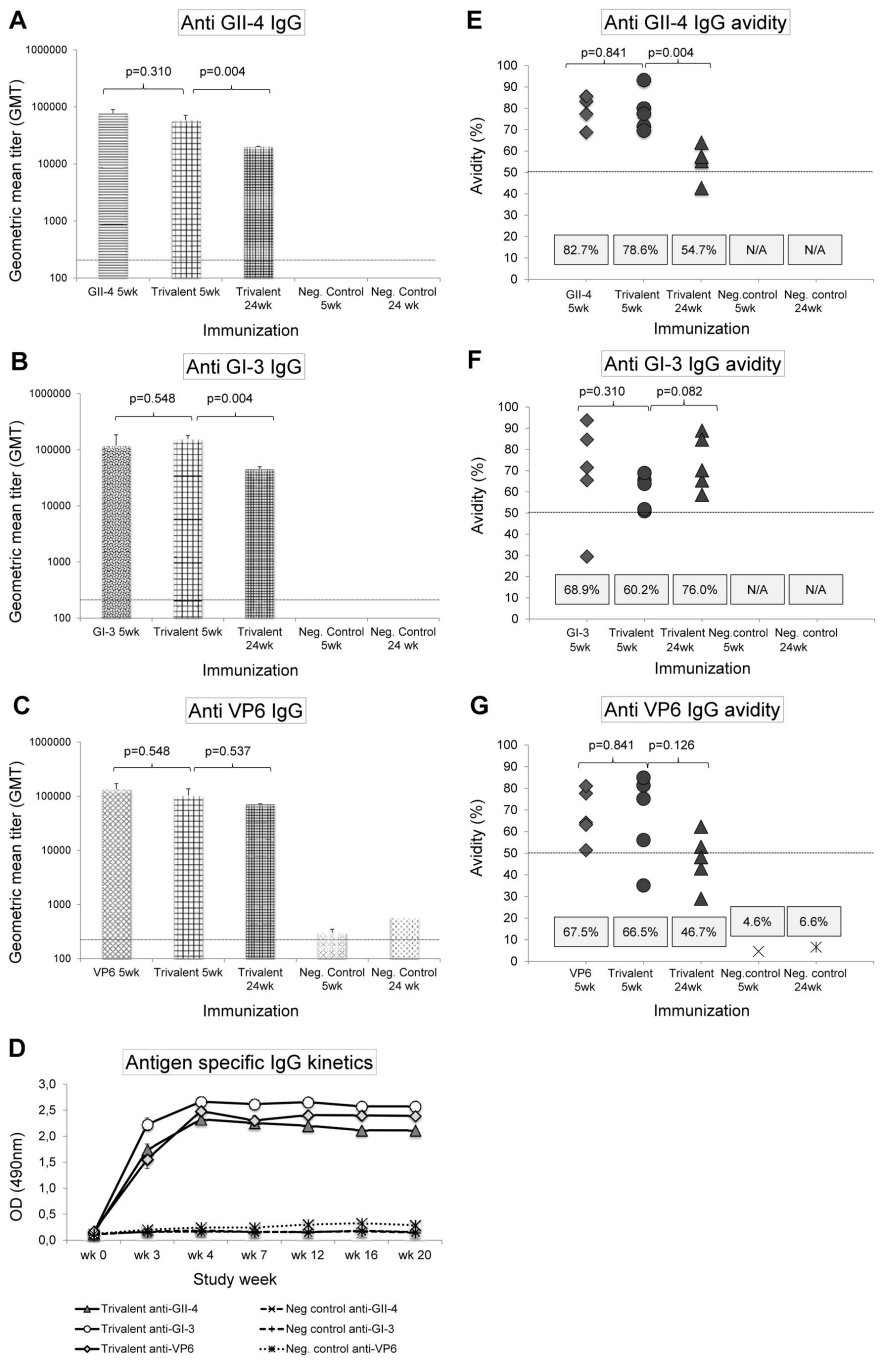
Serum IgG responses induced by the single antigens versus trivalent combination. Mice were immunized two times with 10 µg of the single antigen (GII-4 VLPs, GI-3 VLPs or rVP6) or the trivalent combination (each antigen at a 10 µg dose), and the sera at study week 5 and 24 were tested against GII-4 VLPs (A), GI-3 VLPs (B) and rVP6 (C) in ELISA. Shown are the geometric mean titers (GMTs) of the sera with standard errors of the means. The horizontal lines on panels A-C indicate the limit of detection for the assay. Kinetics of GII-4, GI-3 and VP6-specific IgG responses induced by the trivalent vaccine were measured from tail blood samples of immunized and control mice, and the OD values representing the quantity of antigen-specific IgG at any given time point are shown (D). The avidity of GII-4 (E), GI-3 (F) and rVP6-specific (G) serum IgG antibodies was tested from individual mouse termination sera (at 1:200 dilution) in a modified ELISA in which urea was used to strip off the low avidity antibodies. Shown are the individual mice antigen-specific avidity indexes (%) and the boxed values at the bottom of each figure indicate the group mean avidity indexes. The avidity index was calculated as (OD with urea/OD without urea) × 100%. Avidity index ≥ 50% was considered high avidity. Statistical differences between any two experimental groups were determined by a Mann–Whitney *U*-test and the p-value ≤ 0.05 was considered a statistically significant difference.

Groups of BALB/c mice were immunized two times with 10 µg of NoV GII-4 VLPs, GI-3 VLPs or RV rVP6 as single antigens or with the combination of all three proteins (10+10+10 µg) and the immune responses induced in each group were compared at study week 5. The duration of the immune response induced by the trivalent formulation was followed in another group of mice terminated at study week 24. All antigens induced a robust homologous IgG response (Figure 3A–C) and there were no statistical differences between the immune responses induced by single antigens versus the trivalent formulation (all p>0.05). Although approximately one-fold decrease in the NoV-specific titers occurred from week 5 to 24 (Figure 3A-B), the magnitude of the response still remained high with GII-4 and GI-3-specific titers of 4log10. Kinetics of GII-4, GI-3 and rVP6-specific IgG measured from tail blood samples showed that after the second immunization (at week 3) there were no variations in the levels of antigen-specific IgGs up to study week 20 (Figure 3D). The antigen-specific IgGs were of high avidity (mean avidity index >50%) and no statistically significant differences (p>0.05) were observed between the single versus trivalent combination immunizations (Figure 3E–G) at study week 5. The avidity was long-lasting as high avidity IgGs against all three antigens were still observed 24 weeks after the last immunization in the majority of mice sera receiving trivalent formulation (Figure 3E–G). Antigen-specific IgG subtype titers for IgG1 (representing a Th2 response) and IgG2a (representing a Th1 response) were also measured (data not shown) and Th2/Th1 ratios determined. Trivalent immunization resulted in GII-4, GI-3, and rVP6-specific Th2/Th1 ratios of 0.5, 0.6 and 0.8 respectively, indicating that the combination vaccine triggers a well-balanced Th2/Th1 response.

### Cross reactive antibody responses

**Figure 4:**
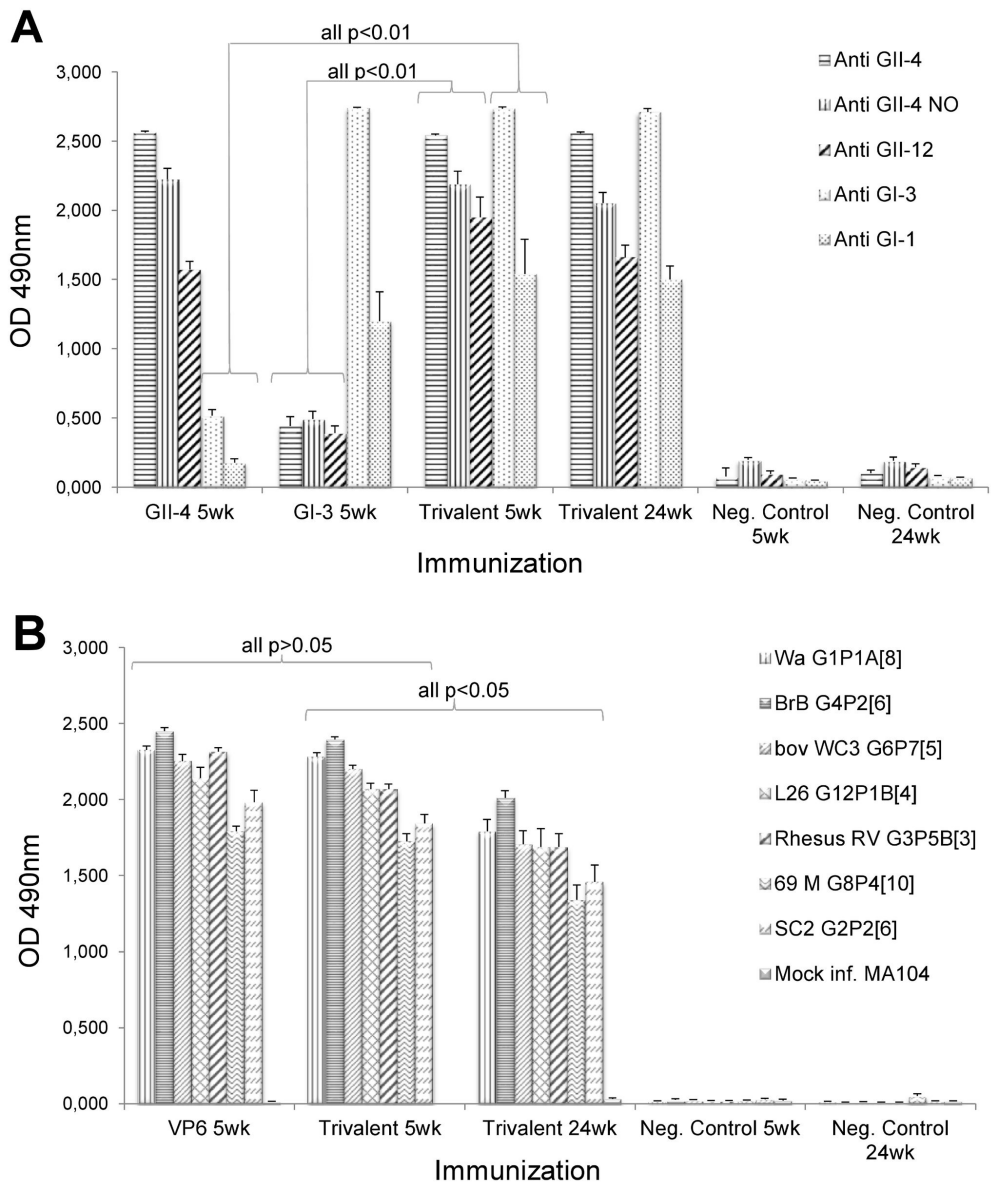
Cross-reactive serum IgG antibodies. Mice were immunized two times with 10 µg of the single antigen (GII-4 VLPs, GI-3 VLPs or rVP6) or the trivalent combination (each antigen at a 10 µg dose) and the sera were tested against heterologous NoV VLPs (A) and RV cell culture antigens (B) in ELISA. Shown are experimental and control groups’ mean OD values representing the quantity of antigen-specific IgG. The error bars represent standard errors of the mean. A Mann–Whitney *U*-test was used to determine statistical differences between single antigen-induced IgG quantities compared with trivalent vaccine induced IgG quantities at study week 5 and IgG quantities induced by the trivalent vaccine at study weeks 5 and 24. The p-value ≤ 0.05 is considered a statistically significant difference.

The cross-reactivity of the serum antibodies induced by the single versus trivalent immunizations were measured in ELISA against heterologous NoV VLPs derived from genogroup II (GII-4 NO and GII-12) and genogroup I (GI-1) not included in the immunization. GII-4 and GI-3 VLP immunizations induced high levels (mean OD>1.5) of cross-reactive antibodies against VLPs belonging to the same genogroup and significantly lower levels (mean OD<0.6, p<0.01) of antibodies against the VLPs belonging to the other genogroup (Figure 4A). The trivalent vaccine immunization triggered high levels of cross-reactive IgGs to all NoV VLPs tested, therefore indicative of a strong humoral response generation against both genogroups of NoVs. In addition, similar levels of intra genogroup antibodies (all p>0.05) were observed in the trivalent combination immunized group compared with the group of VLPs immunized separately, indicating that there was no mutual inhibition of the antigens in the combination (Figure 4A). These cross-reactive NoV-specific IgGs were also of long duration (Figure 4A). In addition to the serum IgG levels represented by the OD value, the GMTs were determined for each study group to confirm the results of the magnitude of cross-reactive IgG response. Similarly to the OD values, the GMTs of cross-reactive antibodies were higher (16 to 32-fold higher, p<0.05) in the trivalent than single immunized mice groups when considering inter genogroup responses (data not shown).

Cross-reactive antibodies against seven RV cell culture antigens belonging to human (G1PA [8], G4P2 [6], G2P2 [6], G8P4 [10] and G12P [4]), bovine (G6P7 [5]), and rhesus RV strains (G3P5B [3]) were detected in mice sera after rVP6 immunization (Figure 4B). No difference in the antibody levels (p>0.05) were noted whether rVP6 was administered alone or in the trivalent combination with NoV VLPs. The magnitude of the response was somewhat lower at the week 24 than at week 5 but still high levels of cross-reactive antibodies (mean OD 1.3-2.0) were detected (Figure 4B).

### Mucosal antibodies and serum VP6 specific IgA

**Figure 5:**
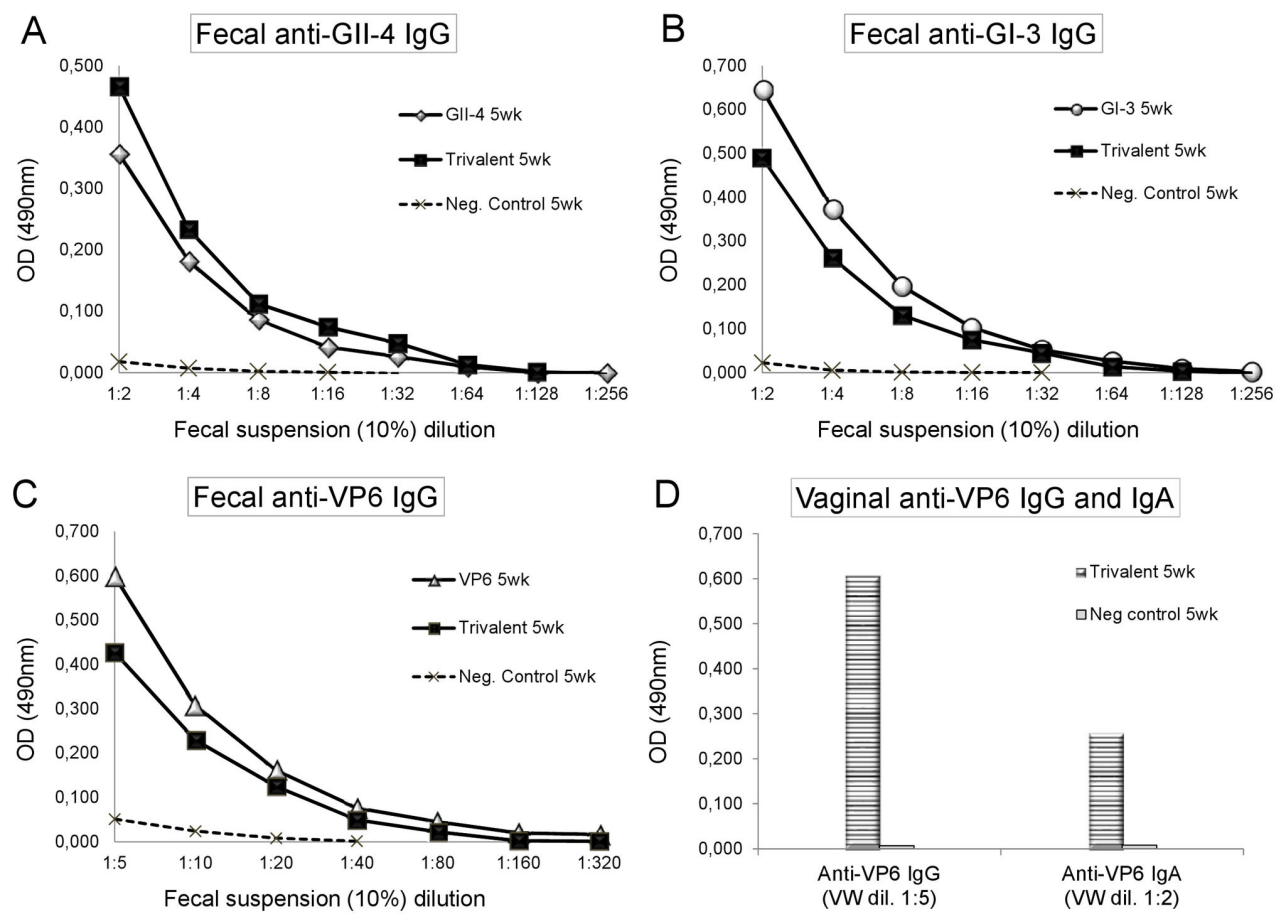
Mucosal antibody response. Group-wise pooled stool samples (10% suspension) of mice immunized with the single NoV GII-4 VLPs, GI-3 VLPs or RV rVP6 antigens or the trivalent combination vaccine were titrated two-fold and anti-GII-4 (A), anti-GI-3 (B) and anti-VP6 (C) IgG content was measured in ELISA. Anti-VP6 IgG and IgA antibodies were measured from the trivalent combination vaccine immunized and control mice vaginal wash samples diluted 1:5 for IgG detection and 1:2 for IgA detection (D). Shown are experimental and control groups’ mean OD values representing the quantity of antigen-specific antibody.

Intestinal NoV and RV-specific IgG were measured from group-wise pooled 10% fecal suspensions in ELISA. Moderate levels of antigen-specific intestinal anti-GII-4 IgG (Figure 5A), anti-GI-3 IgG (Figure 5B), and anti-VP6 IgG (Figure 5C) were detected after each antigen immunizations alone or in the trivalent combination. The stool suspensions from the negative control mice were all IgG negative (Figure 5A–5C). VW samples at study week 5 from the mice immunized with the trivalent combination were tested in ELISA for the detection of RV-specific IgG and IgA antibodies. A moderate level of rVP6-specific IgG and a low level of rVP6-specific IgA were detected from VW samples (Figure 5D). A low level of VP6 specific IgA (OD 0.176, at a 1:2 dilution) was detected from the trivalent combination immunized mice serum (data not shown).

### NoV blocking assays and RV inhibition assay

**Figure 6:**
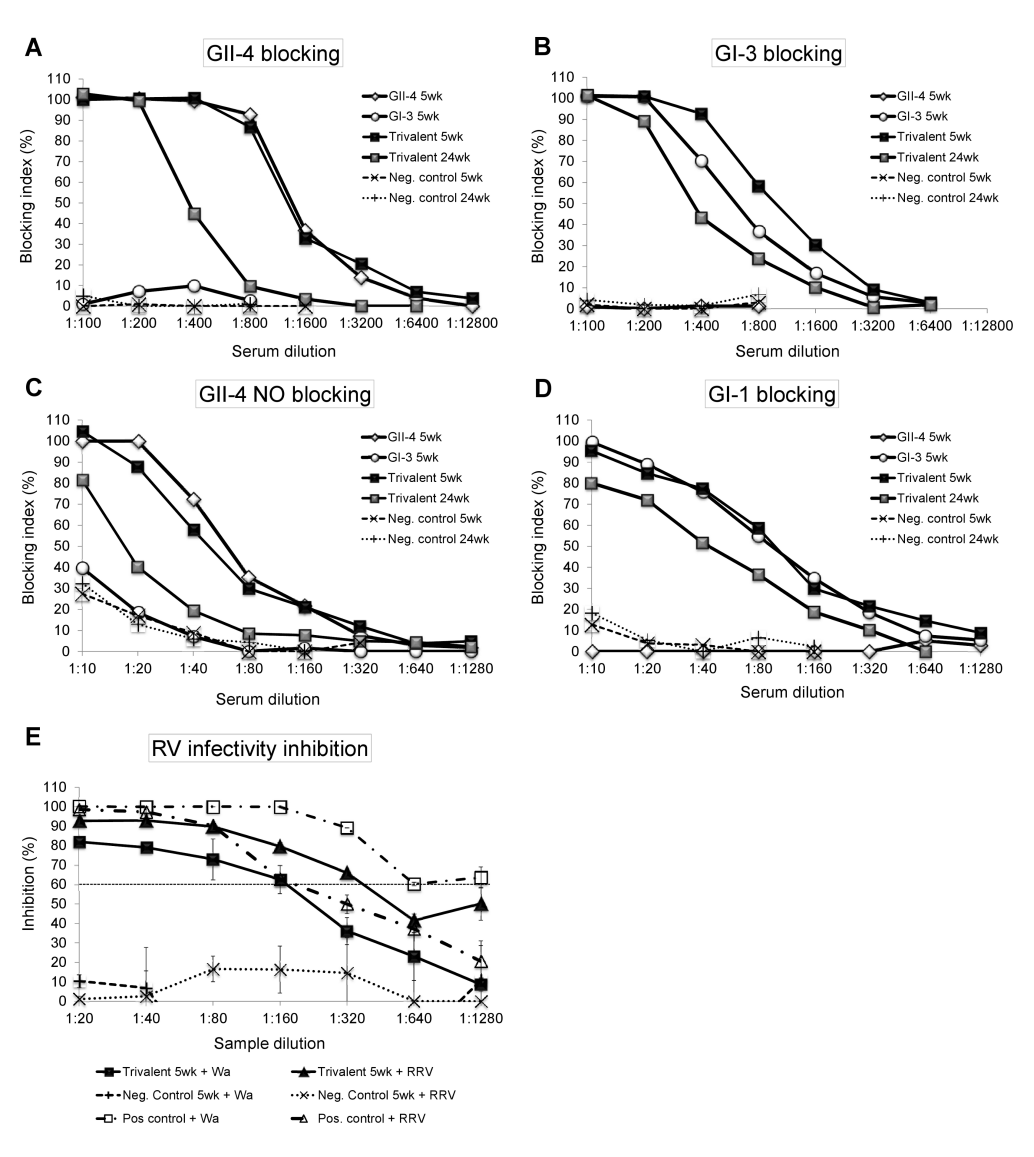
Functionality of NoV and RV-specific antibodies. Termination sera of mice immunized with the single NoV GII-4 or GI-3 VLPs antigens or the trivalent combination vaccine were pooled group-wise, titrated two-fold and used for blocking the binding of homologous GII-4 and GI-3 VLPs (A, B) or heterologous GII-4 NO and GI-1 VLPs (C, D) to human secretor positive saliva (type A for GII-4, GII-4 NO and GI-3 binding and type O for GI-1 binding). Serum from mice receiving the carrier only (PBS) was used as a negative control. The blocking index (%) was calculated as 100% – (OD wells with serum/OD wells without serum, maximum binding) × 100%. Vaginal washes of mice immunized with the trivalent combination vaccine were tested for inhibition of human RV Wa (G1P1A [8]) strain homologous to the immunizing rVP6 protein, or rhesus RV (G3P5B [3]) infectivity by neutralizing ELISA (NELISA). Vaginal washes of mice receiving the carrier only (PBS) and serum from a RV seropositive human donor were used as negative and positive controls. Results are shown as the mean percentage (%) inhibition of rotavirus infectivity of duplicate wells with standard errors. A dashed horizontal line indicates 60% reduction in virus infectivity.

Saliva blocking assays were conducted to study blocking of homologous (immunogen-specific) and heterologous (non-immunogen-specific) NoV VLPs binding to the saliva HBGAs with mice antiserum (Figure 6). Group-wise pooled sera of mice immunized with the single antigen or the trivalent combination blocked homologous GII-4 (Figure 6A) and GI-3 (Figure 6B) VLP binding to saliva HBGAs with a similar intensity. The serum titers for total (100%) blocking of the homologous VLPs binding to the saliva were at maximum 1:400 for GII-4 and 1:200 for GI-3 VLPs. However, mice sera immunized with the GI-3 VLPs alone did not cross-block binding of GII-4 to the saliva (Figure 6A). Likewise, sera of mice immunized with the GII-4 VLPs alone did not cross-block GI-3 VLP binding (Figure 6B). These results indicate that NoV cross-genogroup blocking activity cannot be induced with a single NoV VLP immunization, although cross-reactive binding antibodies were detected in ELISA (Figure 4A). The trivalent combination immunized mice sera were able to block both of the VLPs binding with a similar intensity as the single VLPs immunized mice, and these activities were preserved for the whole 24-week study period (Figure 6A and 6B). Serum blocking of non-immunogen GII-4 NO VLPs (Figure 6C) and GI-1 VLPs (Figure 6D) binding to the saliva was also obtained genogroup-wise; GII-4 immunization induced GII-4 NO and GI-3 immunization GI-1 blocking antibodies. The heterologous blocking activity against VLPs inside the genogroup was similar whether the antigen was administered alone or in the trivalent combination vaccine (Figure 6C and 6D) and lasted until study week 24.

To detect the functionality of VP6-specific antibodies fecal suspensions, VWs and sera were used to inhibit RV infectivity *in vitro* by ELISA-based antigen-reduction neutralization assay [42,43]. Our attempts to use fecal suspensions in the assay failed, probably because of the toxicity of the suspensions for MA104 cells, as previously shown by others [46]. Therefore, we used VWs instead, which likewise to fecal suspensions, contain mucosal antibodies as described above. Inhibition of the infectivity of RVs Wa (G1P1A [8]), homologous to the immunizing protein and RRV (G3P5B [3]), was detected with the VW of the trivalent combination immunized mice with maximum neutralizing titers of 1:160 and 1:320, respectively (Figure 6E). The VW samples from negative control mice did not inhibit RV infection, whereas the positive human control serum neutralized both viruses (Figure 6E). In addition, mouse immune sera did not inhibit RV infectivity *in vitro* (data not shown). The experiments were repeated several times with consistent results.

### Cell mediated immune responses

**Figure 7:**
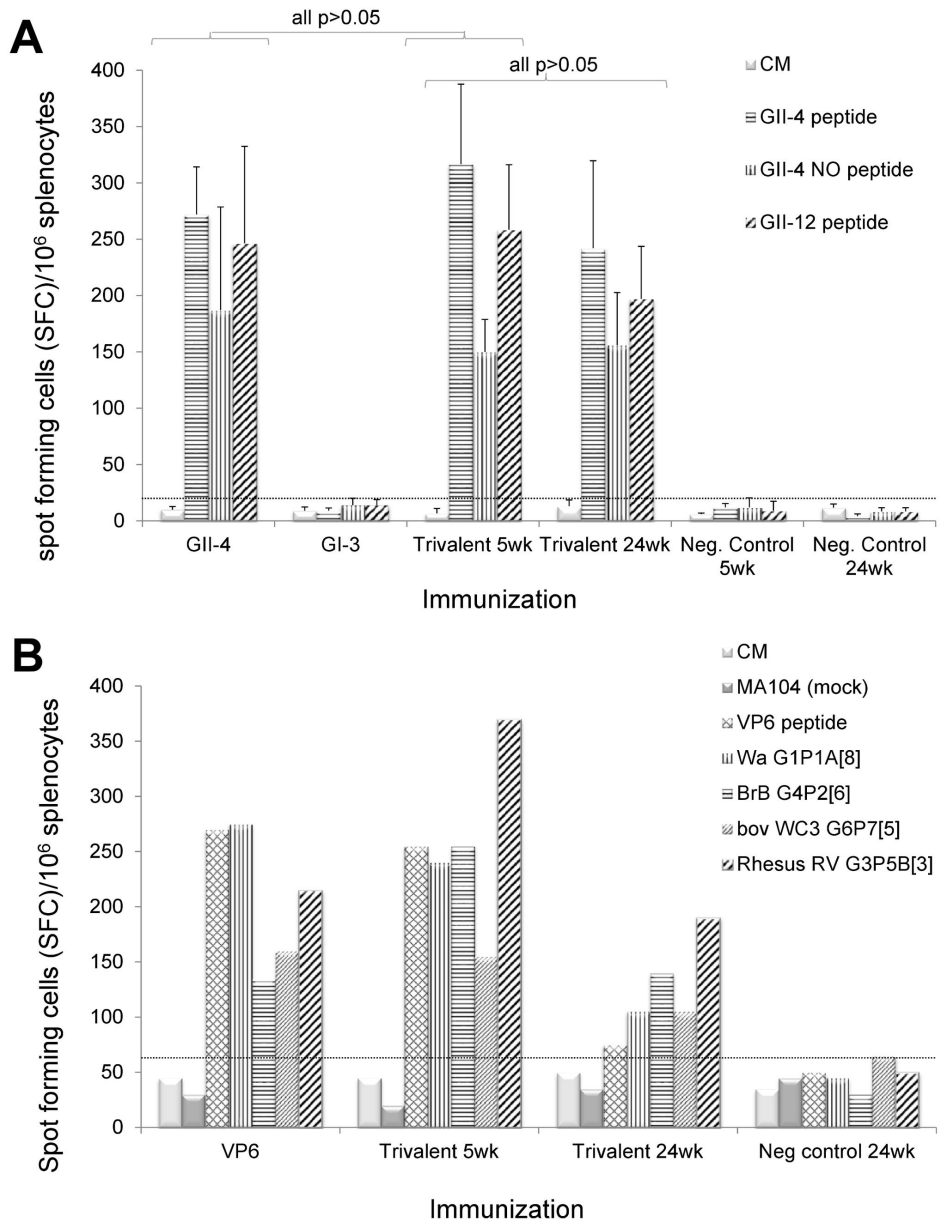
NoV and RV-specific IFN-γ responses. Splenocytes of mice immunized with the single NoV GII-4 or GI-3 VLPs or the trivalent combination vaccine were stimulated with synthetic NoV capsid-derived 15-mer peptides from different NoV genotypes and analyzed for IFN-γ production by an ELISPOT assay (A). The mean spot forming cells (SFC)/10^6^ cells are shown. The error bars represent the standard errors of the mean. The statistical differences between any two experimental groups’ response to a given peptide were determined by a Mann–Whitney *U*-test and the p-value ≤ 0.05 was considered a statistically significant difference. Splenocytes of rVP6 or the trivalent vaccine immunized mice were pooled group-wise and stimulated with synthetic VP6-derived 18-mer peptide or RV cell culture antigens and analyzed for IFN-γ production by the ELISPOT (B). Splenocytes from mice receiving the carrier only (PBS) were used as negative control cells. The mean spot forming cells (SFC)/10^6^ cells of the replicate wells are shown. The dashed line in each figure indicates the maximum background level (cut-off limit) obtained from cells incubated in a culture media (CM) only (mean SFC/10^6^ + 3× SD).

NoV and RV-specific IFN-γ producing cells were quantified from mice splenocytes by an ELISPOT assay (Figure 7). Mice immunized with the GII-4 VLPs or the trivalent combination vaccine elicited a robust IFN-γ response when stimulated with the 15-mer peptides representing capsid P-domain T-cell epitopes [44] derived from homotypic GII-4 or heterotypic GII-4 NO and GII-12 genotypes as described in Materials and Methods. No statistically significant difference was observed in any responses between these experimental groups (p>0.05) at study week 5 (Figure 7A). The IFN-γ response induced by the trivalent vaccine did not diminish over time as IFN-γ producing cell frequency was similar (p>0.05) at study week 5 and 24. GI-3 VLP immunization did not induce any cross-reactive IFN-γ responses to any of the GII peptides. No IFN-γ responses were detected to any peptides by the cells of negative control mice. Immunization with rVP6 either as a single antigen or in the trivalent combination resulted in considerable IFN-γ production when the cells were stimulated with the synthetic peptide representing CD4+ T cell epitope [45] or RV cell culture antigens Wa G1P1A [8], BrB G4P2 [6], bov WC3 G6P7 [5] and rhesus RV G3P5B [3] (Figure 7B). IFN-γ responses were detected against all stimulants at study week 24 but the magnitude of IFN-γ response was up to 3-fold lower in some instances compared with study week 5. No response to mock-infected MA104 cells was detected in any immunized group (Figure 7B) while cell viability was similar in all groups controlled by Con A stimulation (data not shown).

## Discussion

In our previous work we introduced the concept of vaccinating against NoV and RV by parenteral injection of a dual combination vaccine consisting of NoV GII-4 VLPs and RV rVP6 [7]. In the present study we have included GI-3 VLPs as a representative of GI NoVs in the dual vaccine candidate and generated a trivalent combination in an attempt to develop potentially neutralizing cross-reactive antibody responses against GI and GII of NoVs. We also investigated induction of NoV and RV-specific cell mediated immunity as well as RV inhibition by VP6-specific antibodies.

Genogroup I NoVs are antigenically very well conserved [47] and we have chosen GI-3 genotype in the trivalent vaccine combination as it is an important agent in NoV outbreaks and has been the most prevalent GI genotype in pediatric NoV gastroenteritis in Finland in recent years [3]. We hypothesized that by combining VLPs derived from GII-4, the most prevalent NoV genotype worldwide [14], and GI-3 in a single vaccine would give the substantial amount of cross-reactivity needed from a broadly effective NoV vaccine. Recombinant VP6 protein was selected as a part of the trivalent combination vaccine as numerous studies in animal models have documented the protective role of VP6-specific antibodies and T cells in RV infection [30–33]. Both NoV VLPs and rVP6 tubular structures are optimal for dentritic cells uptake [48,49]. The size difference observed between GII-4 and GI-3 VLPs (~38 nm and ~30 nm, respectively) did not affect the immunogenicity of the VLPs as similar immune responses were induced with both of these particles. Although we did not attempt to identify the reason/s for the VLPs size difference, it may be that different number of VP1 monomers are assembled in a single VLP similarly to observations made by White et al. [50]. In addition, natural amino acid differences in the VP1 proteins may drive different size VLP formation [51].

The results from the present study show that two IM immunizations with NoV GII-4 or GI-3 VLPs, either alone or in the trivalent combination with RV rVP6 without an external adjuvant, induced a strong, long-lasting antigen-specific IgG response in mice. In addition the presence of NoV IgG in the gut lumen as detected in here is considered to be an important mechanism in protection against gut infection [52]. As NoVs have great antigenic diversity and are fast evolving viruses, the antibody response elicited by NoV vaccine should be cross-reactive across GI and GII genogroups [19,53]. Our results show that a robust cross-reactive NoV antibody response against both genogroups was solely achieved by the trivalent vaccine, whereas single vaccinations induced a much stronger intra than inter genogroup antibody response. Virus neutralizing potential of the antibodies is an important correlate of protection [6,40,54]. As the traditional neutralization assay is not an option for NoVs that are not able to grow in cell cultures [9], a surrogate neutralization assay named blocking assay using NoV VLPs and HBGAs has been developed instead [41,55]. We have detected high titer of type-specific blocking antibodies in the sera of immunized mice and each antisera was able to block binding of the heterologous VLPs not included in the immunizing formulation but belonging to the same NoV genogroup, namely GII-4 NO and GI-1 VLPs. However, neither GII-4 nor GI-3 VLP immunization alone could induce blocking antibodies towards the VLPs from the other genogroup although cross-reactive binding antibodies were induced (Figure 4A). These observations are in line with the previous findings showing that blocking antibodies are genogroup specific and there is very little inter genogroup blocking activity [47,53,54]. When GII-4 and GI-3 VLPs were combined in the trivalent vaccine, the mice antiserum could block binding of the immunizing and non-immunizing VLPs from both genogroups. The data obtained herein further supports the hypothesis that only multivalent NoV vaccination will induce broadly protective NoV immunity [17–19,53].

The research involving NoV immunity has been largely focused on the antibodies however, cell-mediated immune responses might be important in the clearance of NoV, as has been shown for other viruses [56,57]. We have detected that T cells in the immunized mice produce high levels of IFN-γ in response to synthetic peptides representing T cell epitopes derived from the immunizing (GII-4) [44] and heterotypic (GII-12 and GII-4 NO) NoV genotypes. Lindesmith and co-workers [58] have shown that T cell responses (specifically IFN-γ and IL-2 production) might have been associated with protection in NoV challenge study.

Due to the highly conserved nature [28] RV VP6 protein could provide protection against a broad range of RV serotypes. Although VP6 does not induce serum neutralizing antibodies it has been suggested that VP6 confers protection in mice by inducing a strong CD4+ T-cell response [59] and/or by stimulating mucosal antibodies, especially IgA [60–62]. Our results show that rVP6 assembled in tubular forms is very immunogenic in mice, stimulating a robust, long lasting, high avidity IgG response in serum reactive with various RV strains. Anti-rVP6-specific IgG and IgA were also found in the mucosal samples indicating that an anti-VP6 antibody was being transferred to the gut, the location where the first line of defense is taking place. These mucosal VP6-specific antibodies in contrast to the serum antibodies, inhibited human and rhesus RV infectivity *in vitro*, indicative of the heterotypic protective antibody induction against RVs. Although the mechanism of inhibition remains to be determined, we believe that VP6-specific mucosal IgG and especially IgA are responsible for the inhibition. To support of this, although high level of VP6-specific IgG and low level of VP6-specific IgA were present in serum as well, it did not inhibit RV infectivity. Others have shown that RV VP6 protection from RV infection in vitro and in vivo was mediated by the VP6-specific mucosal IgA and not the VP6-specific serum antibodies [60–64]. Although IM immunization usually elicits systemic immune responses without decent mucosal immunity, it has been shown [65] that naïve B cells acting as antigen presenting cells (APC) are responsible for RV-specific IgA production in the gut after parental immunization in mice. After IM inoculation these APC migrate from draining lymph nodes to mucosal lymphoid tissue, where they induce the production of virus-specific IgA secreting cells. Indeed, Parez and co-workers [66] have shown that specifically RV VP6 protein interacts with a large fraction of naïve B cells via surface immunoglubulins.

We also observed that the cellular immune responses were activated upon rVP6 immunization as the cells of immunized mice produced IFN-γ when stimulated with the VP6-derived peptide representing CD4+ T cell epitope [45] or with various RV cell culture antigens. In our earlier work we have identified CD4+ T cells as being the principal lymphocyte population accountable for IFN-γ production [67]. McNeal and co-workers have shown that CD4+ T cells as the ones we describe here, are the only lymphocyte population responsible for the protective immunity against murine RV [31].

Our results show that the trivalent vaccine consisting of NoV GII-4 and GI-3 VLPs and RV rVP6 1) stimulates strong systemic cross-reactive antibody responses to both viruses with inter NoV genogroup neutralizing ability; 2) induces mucosal antibodies able to inhibit RVs infectivity; and 3) activates the cellular arm of the immune responses to both viruses. Importantly, all the immune responses induced by the trivalent vaccine were long-lasting and no mutual interference and/or inhibition of the vaccine components in the formulation was observed. The results obtained here are encouraging and support the development of a non-live subunit combination vaccine against NoV and RV for humans.
